# Human Skin Models in Biophotonics: Materials, Methods, and Applications

**DOI:** 10.1002/adhm.202501894

**Published:** 2025-07-06

**Authors:** Dardan Bajrami, Fabrizio Spano, Kongchang Wei, Mathias Bonmarin, René M. Rossi

**Affiliations:** ^1^ Swiss Federal Laboratories for Materials Science and Technology Laboratory for Biomimetic Membranes and Textiles Empa Lerchenfeldstrasse 5 St.Gallen 9014 Switzerland; ^2^ School of Engineering Zurich University of Applied Sciences (ZHAW) Technikumstrasse 71 Winterthur 8401 Switzerland; ^3^ Departement of Health Science and Technology ETH Zürich Zürich Switzerland; ^4^ Swiss Federal Laboratories for Materials Science and Technology Laboratory for Biointerfaces Empa Lerchenfeldstrasse 5 St.Gallen 9014 Switzerland

**Keywords:** biophotonics, photonics, skin, skin models, skin phantoms, tissue models

## Abstract

Human skin shows complex optical properties that influence how light is absorbed, scattered, and reflected, playing a key role in biophotonic applications such as diagnostics, imaging, and therapy. Replicating these characteristics in vitro is essential for developing realistic optical skin models that reduce reliance on animal testing and provide standardized platforms for research and product development. This review presents the fundamental optical properties of human skin and outlines the principles and materials used to replicate these properties, including absorbers, scatterers, and matrix materials. Strategies for fabricating optical skin models are discussed, with models categorized according to their structural complexity and functional capabilities. Finally, it explores the state‐of‐the‐art skin models in studying light‐skin interaction and their applications, including optical models that incorporate other physical properties and biological components. With an increase in the usage of light applications on the skin, these models are expected to support the development of personalized biophotonic tools and provide platforms for studying light‐skin interactions under controlled and reproducible conditions.

## Graphical abstract

This review discusses how the optical properties of human skin can be replicated in human skin models. It describes the principles, materials, and techniques used to develop artificial skin for biophotonics research. Finally, the article highlights recent advances and shows how these models improve the study of light‐skin interactions without the need for animal testing.

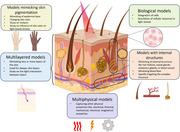

## 1 Introduction

The skin is the largest organ of the human body and functions as an interface between the organism and the external environment. It has important functions such as sensory perception, body thermoregulation, and protection from external threats such as heat, light, mechanical injury, and infection. Structurally, it has three layers: the epidermis, the dermis, and the hypodermis (or fatty layer), representing the outermost, middle, and deepest layer, respectively. Each of these layers has specific physiological and physical properties.^[^ ^1^, ^2^ ^]^ The epidermis, composed primarily of keratinocytes, is a barrier between the human body and the outside world, protecting it from external threats such as microorganisms or UV light. The basal layer of the epidermis contains melanocytes, which produce melanin, a dark pigment responsible for absorbing harmful ultraviolet (UV) light.^[^ ^3^ ^]^ The dermis, on the other hand, is mainly composed of extracellular matrix (ECM), primarily collagen and elastin fibers, with fibroblasts as the main cell type. This layer is responsible for the mechanical strength and elasticity of the skin and contains a complex network of blood vessels, nerves, the lymphatic system, and skin appendages such as hair follicles, sweat glands, and sebaceous glands.^[^ ^4^, ^5^ ^]^ The hypodermis, the deepest layer of the skin, consists primarily of adipose tissue and connective tissue and has an insulating function by protecting against temperature and absorbing mechanical shock.^[^ ^6^ ^]^ In addition to its structural components, the skin has a diverse microbiome that plays a crucial role in immune defense and wound healing and is also an important processor of biochemical processes such as vitamin D synthesis.^[^ ^7^, ^8^ ^]^

Given the complex structure and diverse physiological roles that skin has, in vivo studies on skin are often associated with technical, biological, and ethical challenges. This highlights the growing need for in vitro solutions that can capture the properties of skin in a controlled and reproducible setting. Skin models, or skin phantoms, are synthetic or natural constructs that mimic the properties of human skin. Their ability to mimic physical properties in an in vitro setting under controlled and standardized conditions, as well as their cost‐effectiveness, have shown that they have excellent potential to reduce the need for animal testing, thus enabling a potential revolution in the early stages of product development, research, and education.^[^ ^9^ ^]^ Due to their versatility, skin models are used in numerous scientific fields. In the mechanical field, for example, they are used to study properties such as elastic and viscoelastic properties.^[^ ^10^, ^11^ ^]^ Additionally, they are used to investigate thermal properties, particularly in burn research and thermoregulation studies, where understanding heat transfer in skin layers is crucial.^[^ ^12^, ^13^ ^]^ In the acoustic field, they are used in ultrasound and elastography studies,^[^ ^14^, ^15^ ^]^ while skin models with electric properties are being used in diagnostic imaging techniques.^[^ ^16^, ^17^ ^]^

In the field of biophotonics, which studies the interaction between light and biological tissue, the complexity of the skin is a challenge in the precise investigation of the resulting interactions of the optical processes in the skin. Due to the inhomogeneous composition, the different optical properties of the different skin layers, and the changing physiological conditions, in vivo experiments are often difficult to perform for ethical, technical, and reproducibility reasons. Optical skin models have, therefore, shown themselves to be a promising tool for studying these interactions of human skin and light. The models are specifically engineered to mimic the light absorption, scattering, reflections, and transmission characteristics of human skin and, therefore, enable easy analysis. These models can be either physical models presenting specific properties of human skin or models simulating the behavior of light‐skin interactions.

The development of optical skin models has led to advancements in different fields. In optical therapy, for example, they are used to evaluate the effects of laser treatments, photodynamic therapy, or other light‐based dermatological procedures.^[^ ^18^, ^19^ ^]^ Similarly, in diagnostics, optical skin models help in the development and, ultimately, in the calibration of imaging techniques such as diffuse reflectance spectroscopy or optical coherence tomography (OCT).^[^ ^20^, ^21^ ^]^ Beyond medical applications, such models are also used in the cosmetics industry, where they asses the optical effects of skin care formulations and optimize the design of sunscreens and other photoprotective agents.^[^ ^22^, ^23^ ^]^ In parallel to optical skin models, recent years have seen the emergence of electronic skin (e‐skin) technologies that integrate optoelectronic sensors into flexible, skin‐like platforms.^[^ ^24^, ^25^ ^]^ These systems often combine photonic components with electrical, thermal, or mechanical sensing and have found applications in augmented reality and wearable diagnostics.^[^ ^26^, ^27^ ^]^

The success of optical skin models in biophotonics can be traced back to early efforts to mimic the skin's scattering and absorbance properties. Initial models focused on basic tissue phantoms, which were later refined with the integration of more sophisticated materials that closely mimic the heterogeneous structure of human skin.^[^ ^28^, ^29^, ^30^ ^]^ Over the years, a tremendous endeavor has been made to enhance the accuracy and reproducibility of optical skin models, especially with new, improved fabrication technologies and the better performance and properties of materials utilized to simulate the optical properties of skin at different wavelengths. In recent years, the trend in the development of optical skin models has moved more toward customization and adaptability. Advances in 3D printing, biology, and nanotechnology allow more specific models tailored toward the needs of each patient.^[^ ^31^, ^32^ ^]^ These models are now being optimized not only for academic research but also for product research and development. This trend indicates that skin models will be an important part of personalized medicine in the future. They have the potential to improve patient safety, reduce costs, fasten the development of new therapies and diagnostics, and reduce animal testing.

Given the increasing importance of optical skin models and the understanding of the interaction of light with human skin in various scientific and clinical fields, this review provides an overview of the most important developments and concepts related to optical skin models. The focus of this review remains specifically on optical skin models within the ultraviolet‐visible‐near‐infrared (UV–vis–NIR)è range. Optical biosensing systems and photodetectors, including those developed for e‐skin applications, are beyond the scope of this work. The term “light” in this review refers to optical radiation spanning the UV, visible, and near‐infrared (UV‐Vis‐NIR) spectrum.

## 2 Human Skin: Optical Properties

**Figure 1:**
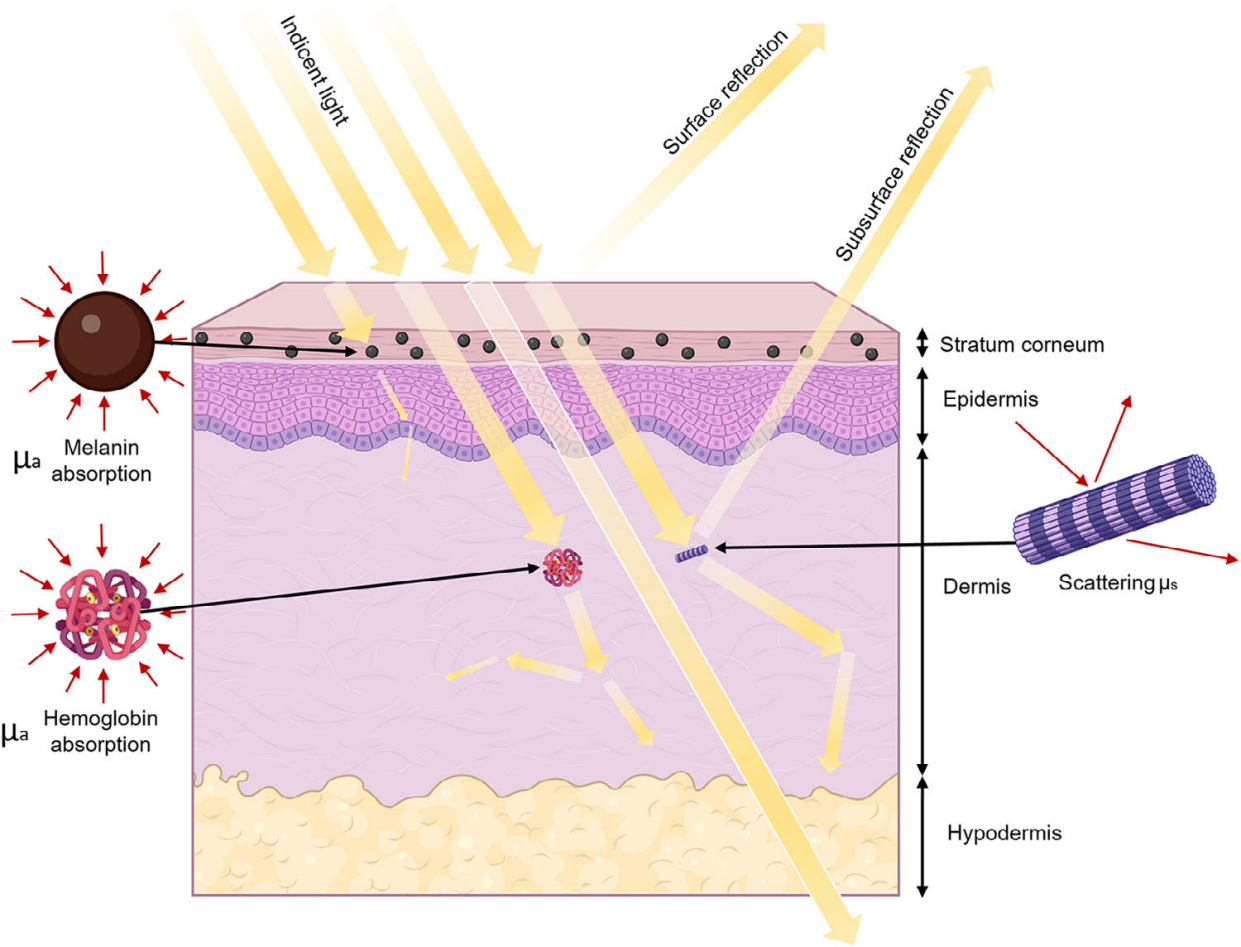
Interaction of light with skin components from different skin layers. Incident light undergoes both surface and subsurface reflection before transmission occurs in the epidermis, where melanin selectively absorbs specific wavelengths, particularly in the ultraviolet (UV) and visible spectrum. In the dermis, hemoglobin is the primary absorber, influencing the spectral characteristics of transmitted light. Light scattering occurs predominantly within the dermis. This is largely due to the fibrous structure of collagen, which, through photon diffusion, redirects the light. The hypodermis is mainly comprised of adipose tissue and contributes insignificantly to absorption while being responsible for light scattering at much deeper levels. The figure illustrates these key optical processes, including absorption, scattering, reflection, and transmission, across different skin layers.

To develop optical skin models, it is necessary to understand the optical properties of skin. However, this is complex because the skin is anisotropic, inhomogeneous, and has various appendages, such as hair follicles, blood vessels, or sweat glands, that interact with the incoming light. In addition, the skin has different chromophores, which are distributed in the different layers and interact dynamically with the incident light across the entire optical spectrum. The most commonly used parameters to characterize the optical properties of skin are the refractive index (n), which quantifies how much the speed of light is reduced inside a medium compared to its speed in a vacuum. The absorption coefficient (µ_a_) quantifies how much light is absorbed per unit depth. The reduced scattering coefficient (µ_s_′) indicates the extent of directional scattering after accounting for anisotropy. Finally, the anisotropy factor (g) describes the average cosine of the scattering angle. These parameters interact because propagation in tissue follows the principles of radiative transfer, with absorption and scattering collectively determining the overall attenuation and directional behavior of light.^[^ ^33^ ^]^ **Figure** **1** illustrates schematically the human skin structure and the light pathway through the skin.

### 2.1 Refractive Index

When light hits the skin, it first hits the sebum layer of the epidermis, which has a higher refractive index (n≈ 1.5) than air (*n* = 1).^[^ ^34^, ^35^ ^]^ The refractive index (n) is a fundamental optical property that determines the speed of light in a particular medium and indicates how light behaves at the interface between different media. It is defined as the ratio of the speed of light in a vacuum (c) to the phase velocity of light (cm) in the medium.^[^ ^33^ ^]^

(1) $$n\left(\lambda \right)=\frac{c}{c_{m}\left(\lambda \right)}$$

Since the refractive index is higher than that of air, a portion of the incident light is reflected at the air‐sebum interface due to the different optical density. The partial reflection follows the so‐called Fresnel equation, which describes the proportion of light that is reflected (R) and transmitted (T) at the interface between two media with different refractive indices. In human skin, this specular reflectance, which results at the air‐sebum interface, can typically account for 4–7% of the incident light (over the spectrum of 250–3000 nm).^[^ ^34^, ^36^ ^]^ Beyond the surface, light that is transmitted into the skin undergoes further refraction due to variations in the refractive indices of different skin layers, including the stratum corneum, epidermis, and dermis.^[^ ^33^, ^37^ ^]^

### 2.2 Absorption

One of the first effects that occurs in the skin tissue is absorption, which is characterized by the absorption coefficient (µ_a_). Light absorption in the skin is determined by the different chromophores in the different skin layers and shows a strong wavelength dependence. In the epidermal layer, melanin, a dark pigment produced in the basal layer, is the main absorber.^[^ ^38^, ^39^ ^]^ The main function of melanin is photoprotection against harmful UV radiation (280–380 nm).^[^ ^40^, ^41^ ^]^ Consequently, light absorption is strongly influenced by skin color, which in turn is determined by the volume fraction of melanin in the epidermal layer.^[^ ^41^, ^42^, ^43^ ^]^ Furthermore, the composition of the different melanin types (eumelanin, pheomelanin), the size of the melanin particles, and their clustering determine the optical absorption properties.^[^ ^40^ ^]^ However, the structure and broadband optical absorption properties of melanin remain incompletely understood.^[^ ^44^, ^45^ ^]^ Other chromophores in the epidermal layer include nucleic acids, urocanic acid, and aromatic amino acids.^[^ ^46^ ^]^

In the dermal layer, absorption is predominantly attributed to hemoglobin (HbA) due to cutaneous blood perfusion.^[^ ^47^ ^]^ In particular, the heme‐iron protoporphyrin IX bound to the polypeptide chains of hemoglobin is responsible for blood absorption.^[^ ^48^ ^]^ Both oxyhemoglobin and deoxyhemoglobin have three absorption peaks. A central peak in the blue region (400–420 nm) and two peaks in the green‐yellow region (540–600 nm).^[^ ^49^ ^]^ Other absorbers inside the dermal layer, like bilirubin, carotenoids, or water, have lower absorption capacity than melanin.^[^ ^48^, ^50^ ^]^ The absorption of light inside the subcutaneous layer is negligible.^[^ ^36^ ^]^ Based on collected and combined in vivo data across Fitzpatrick Skin Types, the reported average µ_a_ ranges from approximately 0.02 to 0.98 mm⁻¹ for FST I‐II, 0.03 to 1.40 mm⁻¹ for FST III‐IV, and 0.03 to 1.25 mm⁻¹ for FST V‐VI across the 400–1000 nm spectrum. The variability of the different studies is substantial and can have average differences between minimum and maximum up to 81% within FST I‐II, 51% in FST III‐IV, and 38% in FST V‐VI, even at the same wavelengths.^[^ ^51^ ^]^ The variability comes mainly from the measurement methodology and biological variability between subjects.

### 2.3 Scattering

Scattering is another significant optical characteristic within human skin. It describes the phenomenon whereby electromagnetic light is deflected due to microscopic fluctuations in refractive indices.^[^ ^52^ ^]^ Scattering effects are crucial and significantly determine the optical pathway, particularly in the dermal layer.^[^ ^48^ ^]^ Scattering in the skin includes Mie scattering, which is mainly caused by filamentous proteins such as keratin in the epidermis layer and collagen fibrils in the dermis. Mie scattering mainly occurs when the wavelength of the incident light exceeds 650 nm, and the struck particles are approximately the size of the wavelength.^[^ ^35^ ^]^ Rayleigh scattering, on the other hand, occurs when particles are significantly smaller than the wavelength of the incident light, leading to scattering in microstructures such as cell nuclei, melanosomes, organelles, or small structures associated with collagen fibrils, and typically occurs up to a wavelength range of ≈650 nm.^[^ ^53^ ^]^ Melanosomes and collagen fibrils are the primary scatterers in the epidermis and dermis layers of human skin. To accurately describe the direction of light scattering in human skin, the anisotropy factor (g) is used, which is ≈0.9 for human skin.^[^ ^48^, ^54^, ^55^ ^]^ The scattering coefficient of in vivo measurements also shows a large variability across studies. Reported average values range from 1.18 to 2.80 mm^−1^ for FST I‐II, 1.04 to 2.09 mm^−1^ for FST III‐IV, and 1.00 to 2.27 mm^−1^ for FST V‐VI within the 400–1000 nm spectrum. Also, here, the differences between maximum and minimum values are high and reach 98% in FST I‐II, 25% in FST III‐IV, and 31% in FST V‐VI.^[^ ^51^ ^]^ Similar to the absorption coefficient, the measurements have high variability due to different skin sides, measurement properties, and subject diversity. In its light pathway in the skin, the light is predominantly scattered or absorbed. The unabsorbed portion of the light exits the skin as diffused reflectance.

### 2.4 Variation of Skin Optical Properties

The physical properties of human skin vary significantly between individuals and across different body regions.^[^ ^56^, ^57^, ^58^ ^]^ The optical properties of the skin, specifically, are highly dependent on factors such as skin thickness, color, hydration, and sebum layer. The variations in these factors are unique to each individual and differ between diverse body regions. For example, the thickness of the skin is influenced by age, gender, ethnicity, and the part of the body.^[^ ^59^, ^60^ ^]^ Recent studies have shown the significant influence of skin color on various optical therapy models, such as oximetry,^[^ ^49^, ^50^ ^]^ wearables,^[^ ^51^ ^]^ and temporal artery thermometers.^[^ ^61^ ^]^ Another example is skin thickness, which affects optical devices due to the scattering properties of the dermal layer. Souza‐Barros et al. have shown that both skin pigmentation and thickness impact the effectiveness of 635 and 808 nm lasers in low‐intensity therapeutics.^[^ ^62^ ^]^ Therefore, to advance optical skin model development, it is crucial to replicate skin properties more individually for a more personalized medicine.

## 3 Replicating Skin: Fundamental Principles and Materials Used

The development of optical skin models enables precise measurements of key skin properties, such as absorption, scattering, and reflectance, and cost‐effective testing of medical products and devices, thus reducing the reliance on animal trials. Nevertheless, the complex structure of skin remains a challenging endeavor. Human skin exhibits a complex internal structure, multiple layers, and anisotropic properties. These features not only determine wavelength‐dependent skin optical properties but also demonstrate substantial inter‐individual variability in appearance, such as pigmentation and moisture content. Additionally, environmental factors like humidity and temperature can affect the optical characteristics. Skin models aim to reproduce the attributes of human skin by replicating specific physical properties, responses to external influences, and structural attributes. A diverse array of skin models has been developed for a large variety of applications.

A common and effective method to replicate skin characteristics is to select a matrix material that defines the core physical attributes, such as optical, thermal, and mechanical characteristics.^[^ ^9^ ^]^ This matrix is a foundation for incorporating refining or additive materials.^[^ ^9^, ^63^ ^]^ The matrix selection process is conducted either individually for each layer^[^ ^64^, ^65^ ^]^ or collectively for all layers, depending on the desired properties.^[^ ^66^, ^67^ ^]^ Subsequently, various materials with specific light‐absorbing, light‐scattering, or other unique physical properties are embedded within particular layers to alter the bulk material's characteristics.^[^ ^9^ ^]^

Optical skin models can be replicated utilizing either synthetic or bio‐based matrix materials. Synthetic materials provide an advantage by developing skin models with greater durability and long‐term reusability.^[^ ^68^, ^69^ ^]^ In recent years, due to their advantages, silicon‐based materials and, in particular, polydimethylsiloxane (PDMS) have emerged as a widespread artificial material. PDMS is cost‐effective, has a long shelf life, and retains its optical properties over extended periods, especially compared to gelatine, which can be used only for a limited amount of time.^[^ ^63^, ^69^ ^]^ Additionally, the solid yet flexible nature of PDMS allows the skin model to be used in both contact and non‐contact applications. Finally, PDMS has a refractive index of ≈1.43, which is very close to that of biological tissue (n≈1.4) and makes it an excellent substitute.^[^ ^63^, ^69^ ^]^ However, the hydrophobic properties of PDMS pose problems, especially when integrating additives. Conversely, biological or bio‐based materials offer a distinct advantage over other materials as they can more closely mimic the properties of human skin, especially collagen and elastin, which are derived from the extracellular matrix (ECM) of human tissue. Nevertheless, they often exhibit inferior durability compared to artificial materials. **Table** **1** presents an overview of the most common matrix materials and their respective advantages and limitations.

**Table 1:** Common matrix materials and their advantages and limitations.

Artificial materials	Advantages	Limitations	References
Epoxy resin	Ease of fabrication Refractive index 1.5 (close to skin)	Rigid	[21, 70, 71]
Aliphatic Polyurethane	Flexible Durable UV‐Resistant	The refractive index does not match human skin	[72]
Polyvyinyl alcohol (PVA)	Biocompatible Water content Flexibility	Limited durability Unstable in water	[18]
Polyvinyl chloride‐polyethylene (PVC‐P)	Transparent	Non‐skin like properties	[73]
Silicone (PDMS, RTV silicone, other silicones)	Customizability Flexibility and elasticity Biocompatibility Low‐Cost Refractive index like skin (n = 1.43)	Hydrophobic	[18, 21, 63, 65, 69, 74, 75, 76, 77]
Bio‐based materials			
Agar	Water content Easy to prepare Biocompatibility Easy to mix with organic compounds	Mechanical properties Dehydration Long term stability	[64, 65, 66, 78]
Gelatin (Gelatin, GelMA)	Water content Modifiable for 3D‐Printing Biocompatibility	Long term stability Dehydration	[20, 64, 66, 67]

Additive materials have the capacity to tailor the optical properties of various skin layers in a wavelength‐specific manner. Additionally, these materials can also add unique optical effects, including, e.g., skin fluorescence. In the matter of optical skin models, absorption, scattering, and fluorescence are the main objectives of replication. The materials utilized, similarly to the matrix materials, originate from both biological and synthetic sources. For absorption recent developments have shown that polydopamine (PDA) may be an excellent substitute for natural melanin due to its similar optical absorption capacity.^[^ ^67^, ^70^, ^79^, ^80^ ^]^ For scattering, Intralipid, an intravenous fat‐based emulsion composed mainly of soybean oil, egg phospholipids, and glycerin, was commonly used as a scattering material.^[^ ^29^, ^81^ ^]^ However, its limited shelf life has restricted its use. Titanium dioxide (TiO2), a white pigment with superior scattering properties, has emerged as the dominant material for mimicking scattering properties.^[^ ^82^ ^]^ **Table** **2** provides a summary of various additive materials, highlighting their respective advantages and limitations.

**Table 2:** Additive Materials.

Absorption Materials (Chromophores)	Advantages	Limitation	References
Coffee	exhibit a strong decay from the visible to near‐infrared (like melanin)	Can vary in concentration, affecting consistency	[65, 66, 69, 83]
Cosmetic skin powder	Color variety	Flat absorption spectra	[74]
Hemoglobin	Absorption in UV and NIR	Limited stability	[20, 49, 53, 78]
India ink	High optical density Easy to use	Limited absorption spectrum	[21, 63, 64, 65, 69, 72, 75, 76]
Nigrosine	Water soluble	Photochemical stability Limited absorption spectrum	[69, 77]
PDA	Scalable in size Similar absorption spectra to natural human skin Broadband absorption Biocompatible	More complex synthesis	[67, 70]
Synthetic melanin	Similar absorption spectra to natural human skin	Possible variability in synthesis	[64, 84]
**Scattering materials**			
Aluminum oxide Al_2_O_3_	High scattering efficiency at shorter wavelengths		[74]
Intralipid®	Consistent, adjustable scattering properties		[64, 78]
Polystyrene beads	Size‐dependent scattering properties		[20]
Titanium Dioxide TiO_2_	Strong scattering, especially at shorter wavelengths		[63, 65, 66, 67, 69, 72, 75, 76, 77, 84, 85]
Zinc Oxide ZnO	Effective scatterer, used for UV‐blocking		[73]
**Fluorescent material (Fluorophores)**			
Flavin adenine dinucleotide FAD	Fluorescence in the blue‐green spectrum Natural fluorescence is essential in cellular metabolism	Affected by environmental factors	[20]

## 4 Skin Models for Biophotonic Applications

In recent years, there has been a rise in the application of light in medicine, with cosmetic laser surgery being an example.^[^ ^86^ ^]^ Consequently, the demand for models that capture the response of human skin to external light stimuli has increased correspondingly for testing, improving, and performing quality control on optical devices. However, as it is difficult to develop a universal model for all applications, capturing all properties of the skin, the scope of optical skin models refers to a specific application area, such as the interaction on skin layers, skin color, or vascularization within a specific wavelength band. Currently, optical skin models have a wide range of applications in nearly all areas of biophotonics.

While simplistic and earlier optical skin models have been designed to focus on a single or narrow range of wavelengths,^[^ ^19^, ^42^, ^73^ ^]^ the goal of more advanced models has been to achieve wavelength dependency and, therefore, to enable the usage of a single model for devices with different wavelength regions. However, the attempt is not straightforward due to the wavelength‐specific dependence of the different chromophores, reflection, and scattering properties. Nevertheless, with more broadband‐responding materials, modern skin models have evolved to become increasingly broadband, mimicking the behavior of human skin across the UV, visible light, and infrared ranges. Recently, a variety of skin models have been developed that can effectively cover a range of wavelengths, ranging from UV to the visible range,^[^ ^70^ ^]^ visible to the infrared range,^[^ ^63^, ^77^, ^87^ ^]^ or nearly the entire wavelength range utilized by optical devices.^[^ ^70^ ^]^

**Figure 2:**
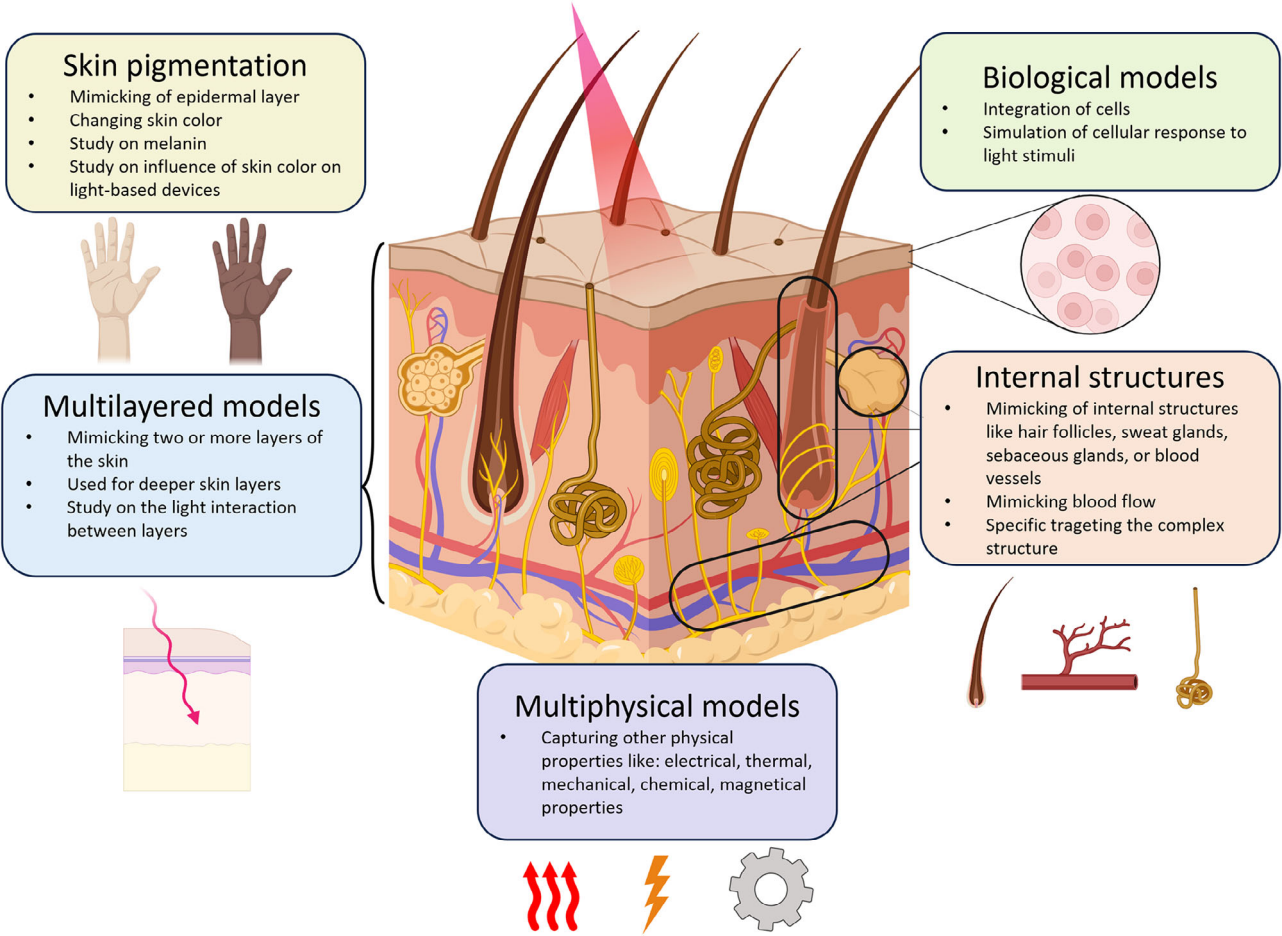
Overview of skin models for biophotonics application: Skin pigmentation models mimic the epidermis, focusing on melanin, skin color variation, and their effects on light‐based devices. Multilayered models simulate multiple skin layers to analyze light interactions across depths. Internal structure models replicate complex anatomical features such as hair follicles, sweat glands, sebaceous glands, and blood vessels. Biological models integrate cellular components to analyze cellular responses to light. Multiphysical models integrate other physical properties, including thermal, electrical, and mechanical characteristics.

In this section, we will explore and discuss the most important applications of optical skin models. **Figure** **2** provides an overview of the different types of skin models used in biophotonics applications. The different types of optical skin models are not strictly subdivided and may overlap between the categories.

### 4.1 Skin Pigmentation

Skin color has a significant influence on the absorption and scattering of human skin and leads to impairments in the treatment or measurements of the skin. For instance, laser hair removal was previously considered more challenging for individuals with darker skin due to the increased risk of injury from laser absorption by melanin. The higher absorption could lead to complications such as post‐inflammatory hyperpigmentation, blistering, and epidermal damage.^[^ ^74^, ^75^ ^]^ Although advancements in laser technology have now enabled hair follicle removal in dark‐skinned individuals, the procedure remains more challenging compared to that for light‐skinned individuals.^[^ ^88^, ^89^ ^]^ Additional research indicates that pulse oximetry measurements in dark‐skinned individuals tend to overestimate SpO2 levels, potentially resulting in the risk of occult hypoxemia.^[^ ^90^, ^91^, ^92^, ^93^ ^]^ These findings underscore the importance of personalized medicine in the treatment of diverse skin colors. Skin models with different skin tones are primarily based on two fundamental applications. First, the influence of melanin distribution, size, and clustering on external light stimuli and the resulting classification of skin color, and second, the specific impact of skin color on biomedical photonics.

Melanin is a complex pigment primarily located in the epidermal layer that provides protection against UV radiation. While the complexity of melanin (e.g., eumelanin, pheomelanin) and its variance within individuals (e.g., size, clustering) have been extensively investigated in various studies on ex vivo skin,^[^ ^94^, ^95^ ^]^ the associated influence on the optical properties of skin has not yet been fully studied. Attempts have been made to replicate the clustering and size range of particles using skin models, with findings compared to ex vivo skin.^[^ ^67^ ^]^ Another significant factor in the treatment of skin is the classification of skin color, which is typically conducted with the Fitzpatrick scale. However, as this classification often relies on the experience and judgment of dermatologists, it can frequently result in an inappropriate selection of lasers, leading to undesired treatment outcomes. Jo et al. have investigated this effect by developing different models correlating to the Fitzpatrick scale and subsequently testing them with a specific laser. The results indicate that the Fitzpatrick scale can only be utilized to a limited extent for the application of lasers and that a detailed classification based on absorbance measurement is necessary.^[^ ^74^ ^]^

Skin models that replicate skin color and are utilized for assessing the influence of various biomedical devices are the main application of pigmented models. These models find utility in a diverse array of specialized fields, including cerebral oximetry,^[^ ^69^ ^]^ 3D‐scanning techniques,^[^ ^53^ ^]^ calibration processes,^[^ ^64^ ^]^ photoacoustic imaging,^[^ ^82^ ^]^ and laser‐based tattoo removal.^[^ ^66^ ^]^

**Figure 3:**
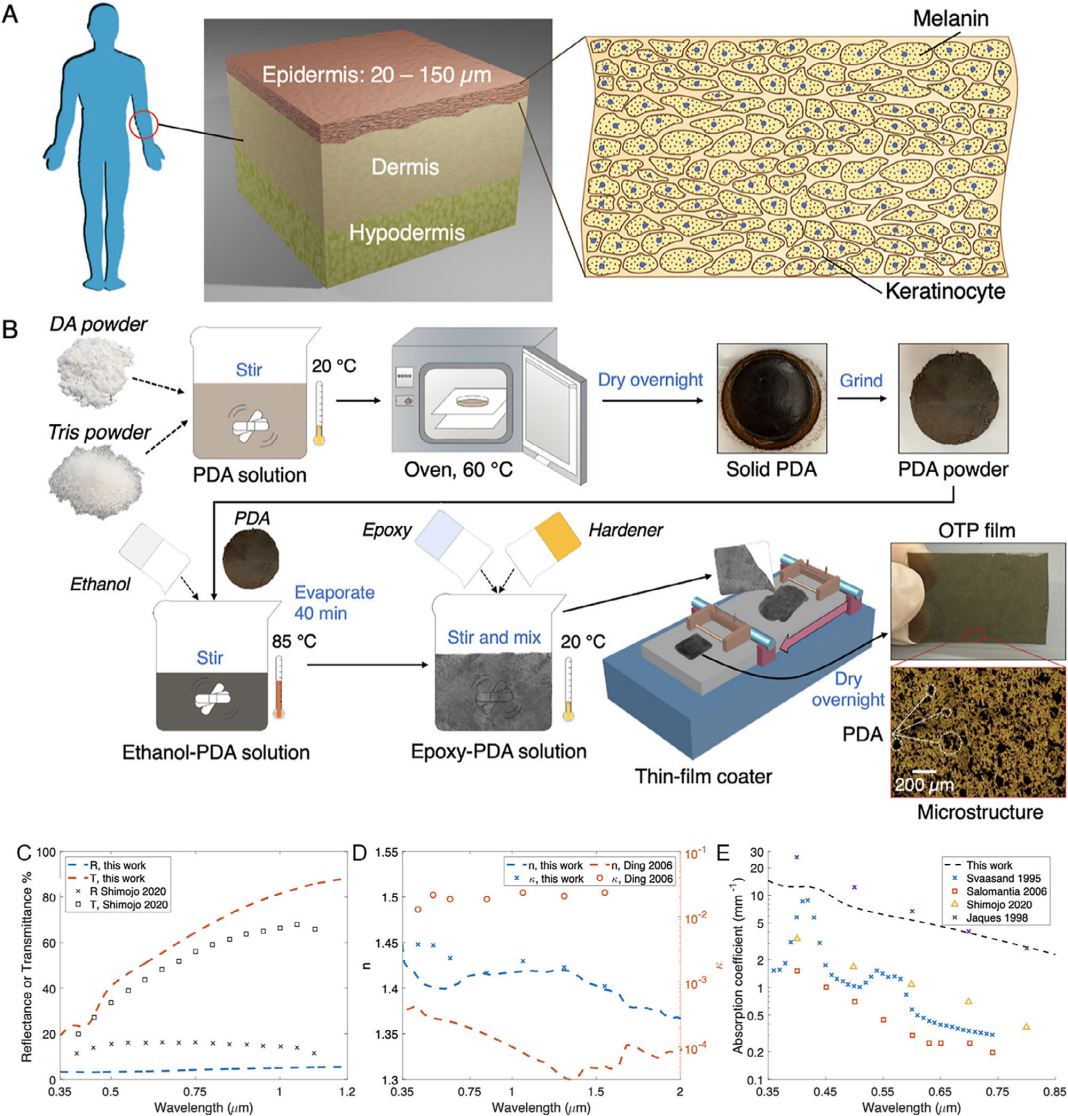
Skin structure and Optical tissue phantom (OTP) fabrication. A) Schematic representation of skin layers, with a detailed view of the epidermis. B) Fabrication procedure of OTP thin films. Comparison of epidermis OTP with published human epidermis results in the UV, vis, and NIR regions. Epidermis OTP and human epidermal tissue comparison for C) reflectance and transmittance, D) complex refractive index, and E) absorption coefficient. Reproduced with permission.^[^ ^70^ ^]^ Copyright 2022, American Chemical Society.

In parallel with the advancement of applications‐specific optical skin models, there has been a notable increase in the development of homogenous epidermal melanin models suitable for a range of applications simultaneously. A notable example is the work of Caratenuto et al.,^[^ ^70^ ^]^ who developed a cost‐effective and easily fabricated epidermis model capable of simulating various skin colors across a broad spectral range from UV to IR (**Figure** **3**). Their approach utilized epoxy resin as a matrix selected for the refractive index similarity to human skin, with polydopamine serving as the absorption medium. The films were barcoated for a controlled thickness (78–152 µm).

The resulting models underwent characterization using UV–vis–NIR spectroscopy, assessing reflectance, transmittance, and refractive index. These properties were then compared to those of ex vivo epidermal tissue. The results showed agreement between experimental and calculated spectra of the epidermal layer, with deviations remaining 10%.^[^ ^70^ ^]^ Particularly for skin with higher melanin content, the models showed strong alignment with real human skin. However, although the phantoms performed well across the UV and visible spectra, they show lower reflectance in NIR region and deviations up to 20% in transmittance in MIR above 1 µm. This could be due to the lack of a scattering agent such as TiO2. Additionally, even though the model was not focused on mechanical properties, the mechanical properties of the epoxy differ from that of natural human skin, leading to potential effects on performance in dynamic applications where skin‐like behavior is required. Such general skin models can be used efficiently and quickly in multiple clinical, research, and commercial applications.^[^ ^70^ ^]^

Pigmentation skin models have made significant progress in mimicking the optical effects of melanin, particularly in the UV to NIR range, with notable improvements using PDA as an absorber. However, they still show limitations in mimicking the heterogeneity of melanin composition (e.g., eumelanin and pheomelanin) and its distribution within the skin layers. Moreover, challenges remain in correlating skin model color with clinical classification systems like the Fitzpatrick scale. Future developments could focus on the integration of more biologically accurate pigment analogs and skin layers that integrate variations in particle type, size, and concentration.

### 4.2 Multilayered Models

The skin is made of different layers, which can be broadly categorized into the epidermis, the dermis, and the hypodermis. These layers perform diverse functions within the skin. The epidermis is a protective barrier against environmental hazards, while the dermis provides structural support, temperature regulation, sensory perception, and aids in wound healing by generating connective tissue. In the later healing phase, the epidermis also contributes by restoring the outer barrier. The hypodermis functions as insulation and shock absorption due to its adipose content.^[^ ^8^ ^]^ These layers also exhibit varying optical properties. While the epidermis is responsible for UV protection and significantly influences the efficacy of therapeutic light‐based devices due to light absorption, the optical properties in the dermis are primarily governed by scattering phenomena. The ability to treat deeper skin layers is crucial for addressing conditions such as angiomas, vascular malformations, or lipomas, and therefore, considering several skin layers is crucial. For example, techniques such as optical coherence tomography (OCT) or laser treatment methodologies are affected by the optical properties of different skin layers. Consequently, various models have been developed that adopt a multilayered approach and show the specific optical properties of each skin layer.^[^ ^20^, ^21^, ^64^, ^65^, ^66^, ^69^, ^71^, ^75^, ^76^ ^]^

**Figure 4:**
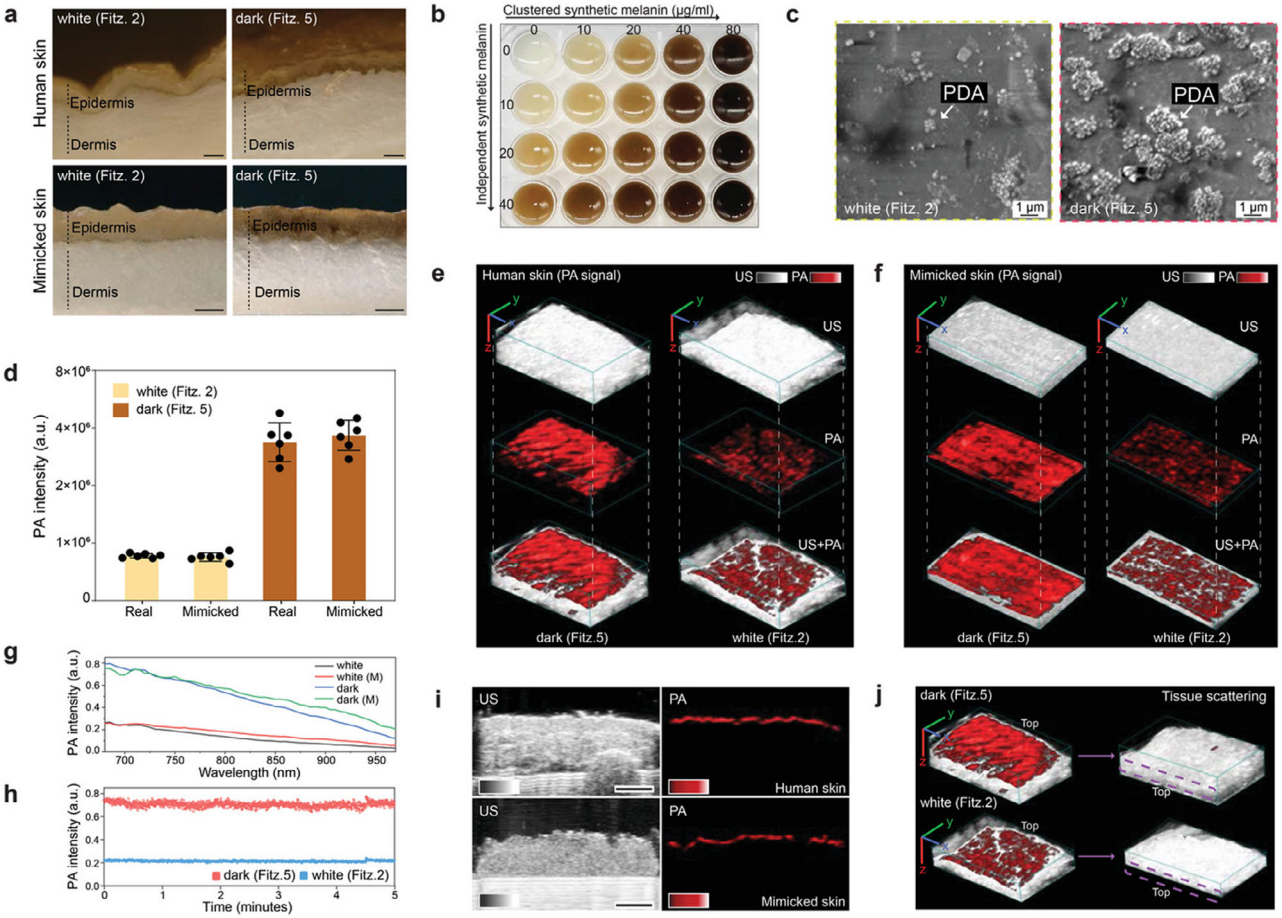
PA comparison between real and mimicked skin. a) Optical images (cross section) of real and mimicked skins. The scale bars represent 0.5 mm. b) Skin phototype variations. Skin colors were readily tunable (From Fitz. 1 to Fitz. 6) by adjusting PDA contents. c) SEM image of PDA mixtures in the printed skin (epidermal layer). d) PA signal comparison between real and mimicked skins. PA signal of mimicked skin was comparable to that of real human skin at the same skin phototype. The error bars represent the standard deviation of six regions of interest. PA and US image of e) real and f) mimicked human skins, showing randomly distributed melanosomes in epidermis. The scale bars (x, y, z) represent 4 mm. g) Spectral PA signal from 680 to 970 nm of real and mimicked (M) skins. h) PA signal stability of mimicked skins. i) PA signal of epidermis in real and mimicked skins. The scale bar represents 4 mm. j) Tissue scattering in real human skins. Purple dotted area indicates the region of epidermis after turning the samples upside‐down. The scale bars (x, y, z) represent 4 mm. The thicknesses of dermal tissue were 4 mm (Fitz. 5) and 2.7 mm (Fitz. 2). The experiments in (g), (i), and (j) were repeated independently three times with similar results. Reproduced with permission.^[^ ^67^ ^]^ Copyright 2022, Wiley.

While previous approaches have made significant contributions to evaluating optical devices and developing skin‐mimicking models, they often face limitations in accurately replicating the complex optical properties of human skin. For instance, earlier models utilizing gelatin with synthetic melanin could not effectively control layer thickness,^[^ ^64^ ^]^ while PDMS silicone with coffee as an absorber did not fully replicate the optical absorption characteristics of real human skin.^[^ ^69^ ^]^ Coffee powder was chosen as an absorbing agent due to its broadband absorption spectrum, which closely resembles that of melanin.^[^ ^69^, ^96^ ^]^ Yim et al.^[^ ^67^ ^]^ used another approach by developing a skin‐mimicking model using 3D bioprinting. Polydopamine (PDA) was utilized as an absorbing material. This combination of PDA and 3D bioprinting enabled precise control over the composition and structure of the skin model (**Figure** **4**a–c), including tunable pigmentation and layer organization. As illustrated in Figure 4d–j, their skin models were analyzed using photoacoustic (PA) testing to assess their optical response, signal stability, and similarity to real human skin across different phototypes. The results demonstrated a direct correlation between skin phototype and light attenuation, with darker skin types absorbing and attenuating more laser energy. This correlation aligns with well‐established optical principles, as higher melanin concentrations result in increased absorption and reduced light penetration.^[^ ^97^, ^98^ ^]^ Furthermore, the new skin model showed stable optical properties under NIR laser illumination, with no degradation over time. Comparisons between real human skin and mimicked skin models showed that the PA signals were comparable, validating the effectiveness of the model.^[^ ^67^ ^]^ Overall, multilayered skin models offer a more optically accurate representation of human skin by separating the high absorption characteristics of the epidermis from the dominant scattering behavior of the dermis, which is not possible with single‐layered models. However, challenges persist in matching optical and mechanical properties at the layer boundaries. Future developments could focus on improving functional stability, particularly for dynamic repeated‐use applications.

### 4.3 Internal Structures

The complexity of the skin goes beyond its layered structure and includes complex internal structures with appendages such as sweat glands, hair follicles, sebaceous glands, and an intricate network of blood vessels in the dermis and hypodermis. These structures perform vital functions important for skin health and appearance. Furthermore, they contribute to the optical properties of skin as they scatter and absorb light. The presence of the appendages significantly affects light penetration, which is essential for the treatment of skin and the specific treatment of the appendages.^[^ ^99^ ^]^ However, their optical effect can often be approximated in bulk tissue models. While their small‐scale structure poses challenges for direct integration, their overall influence on light penetration can still be simulated in simplified models. The need for explicit incorporation of these appendages depends on the application. If the goal is to analyze bulk optical properties, their effects can be mimicked without detailed replication. However, for applications targeting these specific structures, such as imaging or treatment of appendage‐related conditions, their direct integration becomes essential. Consequently, optical skin models require accuracy in representing these elements to support direct treatments on these internal structures, such as port wine stain treatment or laser hair removal. Both treatments rely on selective photo thermolysis, where specific chromophores absorb laser energy, generating heat that selectively damages the target. In port‐wine stain therapy, hemoglobin in abnormal blood vessels absorbs laser energy (typically from a pulsed dye laser), resulting in coagulation of the vessels and gradual removal of the stain.^[^ ^100^ ^]^ In laser hair removal, the melanin in the hair follicles absorbs the laser energy and generates heat that disrupts the follicular structures, preventing hair regrowth.^[^ ^101^ ^]^

The incorporation of vascular structures into optical skin models has been extensively investigated, primarily to develop models for imaging applications. These models aim to mimic the vascular structure of human skin, which enables the validation and calibration of imaging techniques such as optical coherence tomography (OCT) or photoacoustic imaging (PA).^[^ ^64^, ^72^, ^75^, ^102^, ^103^ ^]^ In addition, artificial blood perfusion is simulated by introducing artificial vascular structures into the skin model, which are then perfused with artificial or natural blood.^[^ ^75^, ^83^, ^84^ ^]^ Vasculature is mimicked by embedding artificial tubes, frequently constructed from materials such as PDMS, within the model's structure. Another method involves molding the blood vessels to perfuse them in a second step.^[^ ^84^ ^]^ Recent advancements have also utilized microfluidic cells to simulate smaller vessels and blood flow in the skin. In addition, fluorophores are integrated to simulate fluorescence within the skin. This is achieved by mixing fluorescent materials with a biocompatible matrix material, allowing for a realistic simulation of fluorescence within the model.^[^ ^78^ ^]^

The problem with the integration of appendages lies mainly in the small‐scale structure and the complexity of the geometry, which are difficult to incorporate, and why previous studies have not addressed this issue. The integration of complex structures into skin models has undergone a significant transformation with the emergence of 3D printing technology. Whereas previously, these structures were manually constructed, 3D printing now allows for the direct fabrication of intricated and customized structures within models, improving both precision and accuracy. The layered structure of the skin corresponds well with the additive manufacturing process of 3D printing, making it an optimal approach for developing complex models. Various 3D printing techniques, such as stereolithography (SLA),^[^ ^75^ ^]^ fused deposition modeling (FDM),^[^ ^76^ ^]^ inkjet printing,^[^ ^104^ ^]^ and extrusion‐based bioprinting,^[^ ^66^, ^67^, ^85^ ^]^ have been used for this purpose. With advancements in 3D printing technology, the ability to replicate biological structures is suspected to improve, allowing the integration of complex features such as hair follicles, sebaceous, and sweat glands into these models. Until now, their small size and complex geometry have posed a major challenge to manufacturing, but advances in high‐resolution bioprinting are helping to overcome these limitations.

### 4.4 Multiphysical Skin Models

While multilayered optical skin models primarily focus on replicating the structural and optical properties of different skin layers (epidermis, dermis, hypodermis), multiphysical skin models go beyond this by adding additional physical properties such as thermal, mechanical, and electrical characteristics.

For example, the propagation of electromagnetic waves, thermal effects, and mechanical deformations can play a role in optical systems and influence the interaction of light with biological tissue. In cases such as photothermal therapy, accurate simulation of heat diffusion, together with mechanical and optical properties, is crucial to achieve realistic results. Therefore, various skin models have been developed that take into account additional properties such as mechanical,^[^ ^64^, ^66^ ^]^ acoustic,^[^ ^64^, ^67^ ^]^ and thermal^[^ ^18^, ^105^, ^106^ ^]^ characteristics. To fully understand the behavior of such systems, the interaction of these processes must be taken into account, which can be achieved by hybrid models.

**Figure 5:**
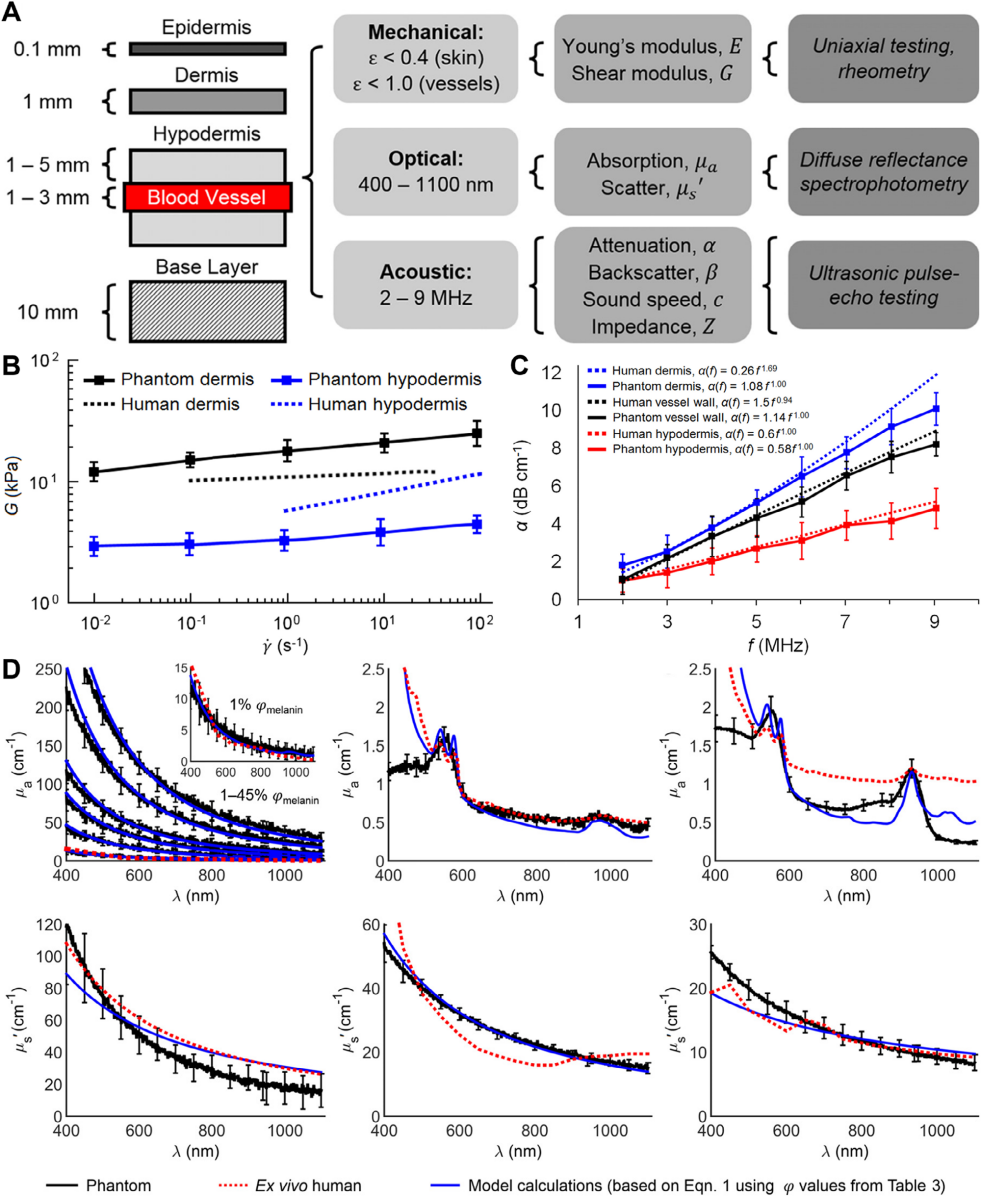
A) Design and characterization of skin and vessel‐mimicking phantoms. Left: Phantoms consist of a 0.1 mm epidermis, 1 mm dermis, and 10 mm hypodermis. Blood vessel substitutes (1–3.2 mm) are embedded at varying depths, perfused with blood‐mimicking fluid. Center: Phantoms replicate mechanical, optical (400–1100 nm), and acoustic (2–9 MHz) properties of human tissues. Right: Material properties were assessed and compared to human data. B) Shear modulus G of phantom dermis (black) and hypodermis (blue), measured via rheological frequency sweep (γ˙ = 10^−2^ to 10^2^ s^−1^), showing human tissue‐like behavior (dotted lines). C) Acoustic attenuation α of phantom dermis, hypodermis, and vessel wall (solid lines) versus human data (2–9 MHz). Power law parameters α1 and nα are provided. D) Optical absorption (µ_a_, top) and reduced scattering (µs, bottom) of phantom and human skin layers. Absorption of phantom epidermis (black) at varying melanin fractions (φmel) versus model calculations (blue) and human data (red dotted). Inset: Absorption for 1% φmel. Scattering profiles of phantom dermis and hypodermis compared to models and *ex vivo* human data. Reproduced with permission.^[^ ^64^ ^]^ Copyright 2016, Wiley.

A notable example is presented by Chen et al., who developed a multiphysical model for applications in multimodal skin and vascular imaging techniques and image‐guided interventions.^[^ ^64^ ^]^ In their work, a multi‐layered model was developed that mimics the mechanical, optical, and acoustic properties of five peripheral tissues: epidermis, dermis, hypodermis, blood vessels, and blood (**Figure** **5**). The model was produced in a 3D‐printed container. The epidermis layer was prepared with gelatin, the dermis layer with 24% gelatin and 1% agar, while the hypodermis was composed of 2% gelatin and 0.2% agar. The layers were sequentially poured into the container and allowed to cool. PDMS tubes were utilized as blood vessels, and blood‐mimicking fluid consisting of water/glycerol/dextran base with 10 µm polyamide microspheres was used to simulate blood. The properties of the skin were modified using synthetic melanin for absorption in the epidermis, India Ink (See Table 2) for absorption in the dermis and hypodermis, Intralipid® for scattering in the dermis and hypodermis, bovine serum albumin (BSA) for acoustic attenuation, and silica microspheres for acoustic backscatter parameters. The models were subsequently characterized using various methods (Figure 5) and compared to *ex vivo* data. The results compared with the in vivo and *ex vivo* data of human skin demonstrate that the Young's modulus, optical absorption, optical scattering, and acoustic attenuation are largely consistent in a qualitative comparison, with a maximum difference of 9.73% for optical scattering in the hypodermis and a minimum difference of 1.06% for Young's modulus for the vessel walls (Figure 5B–D). However, the authors also highlighted the various limitations of their study. The models were partially compared (Young's modulus, optical absorption, and optical scattering) with *ex vivo* data because in vivo data were not readily available. Direct comparison is problematic due to the changes in physical characteristics of the skin after the transplantation. Additionally, a potential evaluation of model stability over several months was noted, which is relevant for the utilization of skin models. Furthermore, the development of custom vessel tubing with alternative materials such as gelatin or agar can enhance the material properties, particularly acoustic impedance. Chen et al. established a foundation of such multiphysical and multilayer models and paved the way for future more precise (e.g., by 3D‐printing) and more accurate (new materials, e.g., polydopamine) models.

Multiphysical skin models are an important step in skin model development due to their ability to capture the interplay of physical processes involved in optical skin treatments. However, challenges remain in precisely mimicking specific physical properties, particularly when integrating materials with differing characteristics, as their interactions can compromise performance. Further research is needed to refine material combinations that maintain both optical precision and other physical properties without mutual interference.

### 4.5 Biological Models

Physical models show limitations in their inability to reproduce the highly dynamic processes occurring in the skin, primarily at the cellular level. While the general optical properties coming from cells can be mimicked using physical models, the specific biological processes themselves, such as proliferation, immune reactions, molecular changes, and cell death, cannot be replicated at the cellular level. Understanding these biological dynamics is important for research, even if their direct optical relevance is limited. Biological models aim to address this limitation by incorporating cells into tissue‐engineered models and subjecting them to optical stimuli. However, the disadvantage of these models is that their physical properties are often far from those of natural skin. In addition, they are not easy to handle and have a shorter lifespan than physical skin models.

In recent years, in vitro biological models have demonstrated significant advancements. Cell cultures that mimic the skin are increasingly complex and can mimic skin in 3D models with different layers, skin color, and cell types.^[^ ^107^, ^108^ ^]^ Different models have been developed to understand the importance of the role of ultraviolet B (UVB) radiation in cancer development^[^ ^109^ ^]^ or its harmful effects on other cells^[^ ^110^ ^]^ in research on the importance of aging processes due to UV radiation,^[^ ^111^ ^]^ the immune behavior of cells in response to a light stimulus,^[^ ^112^ ^]^ or the evaluation of sunscreen efficacy on cells.^[^ ^113^ ^]^ In the future, more straightforward studies, such as those examining the effects of blue light on cells in 2D cultures,^[^ ^114^, ^115^ ^]^ could be translated into more complex 3D in vitro models. While physical models are highly effective for replicating the optical properties of skin, biological models offer a different perspective by allowing the study of cellular responses and dynamic biological processes that physical models cannot capture. However, their main limitation remains short shelf‐life and biological variability. Therefore, the use of biological models to understand physical processes in the skin and the general testing of medical devices is still in its early stages. Still, it could become a relevant topic in the coming years. A hybrid approach combining biological responsiveness with optical reproducibility may address these issues. Rather than replacing one another, these models can serve complementary roles.

## 5 Outlook and Conclusion

To develop optical skin models with a higher level of complexity at the microstructural level, 3D printing technologies are being increasingly used and developed. Tissue‐mimicking models are already using this technology to improve their simulations and better understand the properties of the skin.^[^ ^116^, ^117^, ^118^, ^119^, ^120^, ^121^, ^122^ ^]^ Recent advances in 3D printing technology have made it possible to print coronary artery trees with fluorescent properties^[^ ^103^, ^123^ ^]^ or tumors.^[^ ^124^ ^]^ This breakthrough will make it feasible to print complex structures directly into optical skin models in the near future. Another technology emerging in the 3D‐printing field is two‐photon 3D printing, which enables the printing of microstructures like microvasculature and the future development of even more detailed skin structures.^[^ ^125^ ^]^ Furthermore, it can be expected that future optical skin models will increasingly specialize in individual characteristics, driven by the growing demand for personalized medicine. Multiphysical models, as seen in the case of Chen et al., are taking on more functions and getting closer to replicating the realism of skin.^[^ ^64^ ^]^ From a biological perspective, further specialized in vitro skin models specific to optical applications are anticipated. Moreover, the combination of artificial and biological models may introduce an additional dimension to skin models, where the general optical properties can be consistently simulated by the physical part of the model, while specific analyses of biological processes in the skin can be conducted through the integration of partial biological components. This hybrid approach could aim to balance the strengths of both physical and biological models. Physical models provide stable and reproducible optical properties, making them valuable for standardized optical studies, while biological components allow for a more dynamic investigation of cellular responses and biological interactions. For purely optical studies, physical models may be sufficient, as they effectively replicate key optical parameters without the variability introduced by biological systems. Yet, in research involving biological reactions to light, such as phototoxicity, immune responses, or tissue regeneration, biological integration becomes necessary. Nevertheless, biological models come with limitations, such as shorter lifespans, variability, and handling challenges, making their use less practical for long‐term or large‐scale studies. Moreover, despite the advances in bioengineering, current biological models still have issues replicating the full biological complexity of skin. Critical elements such as functional vasculature, immune‐responsive components, and appendages are rarely included, even though they influence how light interacts with skin. These technological and conceptual advancements suggest a new generation of optical skin models that could be more versatile, realistic, and adaptable to various research needs.

Human skin models have been shown to be valuable tools for demonstrating the interaction between electromagnetic radiation and human skin. The relevance of human skin models lies in their capacity to capture specific physical (and biological) properties of the skin and to use them in research, development, testing, and training, thereby reducing the need for animal trials while advancing our knowledge of human physiology. Moreover, they can reveal multifaceted and interrelated physical interactions within the skin by integrating other physical properties, such as thermal components or mechanical force, and incorporating biological materials. The future of optical skin models appears promising due to the increasing wavelength‐specific adaptation of skin models, the addition of physical properties (multiphysical approach), and the improvement of technologies such as 3D printing, which allow for developing skin models with greater complexity than previous models. However, it is essential to acknowledge the limitations of these skin models. Despite their advancements, they remain approximations and cannot fully replicate the complete physical complexity of human skin. Current skin models do have limitations in mimicking real skin behavior, especially in the UV and MIR ranges. Recognizing these limitations ensures they are applied appropriately in research, medical device testing, and optical studies. Without this awareness, there is a risk of overestimating their accuracy or misapplying them in contexts where their physical properties may not fully match real skin behavior.

In conclusion, this scientific review provides an overview of the diverse applications of human skin models linked to biophotonics applications. In the future, optical skin models are expected to substantially advance our comprehension of human skin and the interaction between light and skin. By allowing more precise simulations of light‐tissue interactions, they directly support the field of biophotonics, enabling innovations in imaging, diagnostics, and light‐based skin therapies. Finally, the advancements in skin models can contribute to the reduction of animal experimentation, which is a crucial aspect of skin model development and aligns with the growing legal and public demand for alternative methods that are more humane and ethically responsible.

## Conflict of Interest

The authors declare no conflict of interest.

## Author Contributions

D.B. contributed to the conceptualization, methodology, formal analysis, investigation, data curation, writing – original draft, writing – review and editing, visualization, and project administration. K.W. and F.S. contributed to conceptualization, writing – review and editing, visualization, supervision, and project administration. M.B. and R.R. contributed to conceptualization, writing – review and editing, visualization, supervision, project administration, and funding acquisition.
